# Serum Levels of Transforming Growth Factor Beta 1 in Systemic Lupus Erythematosus Patients

**DOI:** 10.3390/biom13010073

**Published:** 2022-12-29

**Authors:** Fuensanta Gómez-Bernal, Juan Carlos Quevedo-Abeledo, María García-González, Yolanda Fernández-Cladera, Agustín F. González-Rivero, Antonia de Vera-González, Candelaria Martín-González, Miguel Á. González-Gay, Iván Ferraz-Amaro

**Affiliations:** 1 Division of Central Laboratory, Hospital Universitario de Canarias, 38320 Tenerife, Spain; 2 Division of Rheumatology, Hospital Doctor Negrín, 35010 Las Palmas de Gran Canaria, Spain; 3 Division of Rheumatology, Hospital Universitario de Canarias, 38320 Tenerife, Spain; 4 Division of Internal Medicine, Hospital Universitario de Canarias, 38320 Tenerife, Spain; 5 Department of Internal Medicine, University of La Laguna (ULL), 38200 Tenerife, Spain; 6 Epidemiology, Genetics and Atherosclerosis Research Group on Systemic Inflammatory Diseases, Hospital Universitario Marqués de Valdecilla, IDIVAL, 39011 Santander, Spain; 7 Division of Rheumatology, Hospital Universitario Marqués de Valdecilla, Universidad de Cantabria, 39011 Santander, Spain; 8 Cardiovascular Pathophysiology and Genomics Research Unit, School of Physiology, Faculty of Health Sciences, University of the Witwatersrand, Johannesburg 2000, South Africa

**Keywords:** transforming growth factor beta, systemic lupus erythematosus, disease damage

## Abstract

Transforming growth factor beta (TGF-β) is a highly pleiotropic cytokine that has broad anti-inflammatory and immunosuppressive effects. In patients with systemic lupus erythematosus (SLE), the immunosuppressive effect of TGF-β1 is thought to be dysfunctional. In the present work, we aimed to study the relationship between the serum levels of TGF-β1 with the characteristics of the disease as well as with the patterns of activity, damage, or severity of the disease. Two hundred and eighty-four patients with well-characterized SLE were recruited. The serum levels of TGF-β1 were assessed. A multivariable linear regression analysis was performed to analyze the relation of disease characteristics to TGF-β1. The Katz severity index (beta coefficient 179 [95% confidence interval 7–350] pg/mL, *p* = 0.041) and SLEDAI activity index (beta coefficient 96 [95% CI 20–171] pg/mL, *p* = 0.014) were associated with higher serum levels of TGF-β1 after the multivariable analysis. When the disease-specific features were studied, ocular and cardiovascular manifestations were positively associated with serum TGF-β1 levels. In contrast, gastrointestinal and musculoskeletal involvements were associated with lower levels of circulating TGF-β1. Among patients with SLE, the serum levels of TGF-β1 were highly associated with disease-related manifestations.

## 1. Introduction

Transforming growth factor beta (TGF-β) is a highly pleiotropic cytokine that is an important contributor to the control of apoptosis, angiogenesis, wound healing, immune regulation, and tumor biology. TGF-β exists in three isoforms (TGF-β1, TGF-β2, and TGF-β3), and virtually all cells have receptors for TGF-βs. Immune system cells mainly produce TGF-β1, and this isoform is commonly found in plasma [1]. The immunological role of TGFβ1 includes broad anti-inflammatory and immunosuppressive effects [2], and the complete knockout of TGF-β1 in mice results in autoimmunity and early death from a multi-organ inflammatory syndrome [1]. However, not all of the effects of TGF-β1 are suppressive because, in combination with certain cytokines and T cells, TGF-β1 was also linked to inflammation and autoimmunity [3].

Systemic lupus erythematosus (SLE) is a chronic and multisystem immune-mediated disorder. Patients may present with a wide array of symptoms, signs, and laboratory findings and have a variable prognosis that depends upon the disease severity and type of organ involvement. Although the exact etiology of SLE remains obscure, it is clear that many of the clinical manifestations of SLE are mediated directly or indirectly by antibody formation and the creation of immune complexes. Immune abnormalities that have been described in SLE relate to vicious cycles, including the increase in levels of microparticles, which are small, membrane-bound vesicles that contain DNA, RNA, nuclear proteins, cell-adhesion molecules, growth factors, and cytokines [4]. Furthermore, studies in humans suggest that, in patients with SLE, the expression of TGF-β1 is dysfunctional. In this sense, it is believed that, in SLE, natural killers and T cells fail to release adequate immunosuppressive amounts of TGF-β [5]. In addition, it was described that the production of TGF-β1 by lymphocytes isolated from SLE patients is reduced compared to that of control individuals [6] and that autoantibody production in SLE could be abrogated by treatment with TGF-β [7]. In this sense, the *TGFB1* (rs1800470) variant is independently associated with susceptibility, TGF-β1 plasma levels, and biological activity in patients with SLE [8] and patients that carry certain haplotypes of the *FOXP3* (forkhead box protein P3) gene have higher TGF-β1 levels [9]. In addition, studies have shown that TGF- β1 is required for the lineage commitment of pathogenic IL-17-producing T helper cells [10,11].

In the present work, we assessed the serum levels of TGF-β1 in a large series of well-characterized patients with SLE. We aimed to study the relationship between these serum levels of TGF-β1 with the characteristics of the disease as well as with the patterns of activity, damage, or severity of the disease.

## 2. Materials and Methods

### 2.1. Study Participants

This was a cross-sectional study that included 284 patients with SLE. All SLE patients were 18 years old or older, had a clinical diagnosis of SLE, and fulfilled ≥ 4 American College of Rheumatology (ACR) classification criteria for SLE [12]. They were diagnosed by rheumatologists and were periodically followed up at rheumatology outpatient clinics. Patients taking prednisone at an equivalent dose of ≤10 mg/day were allowed to participate, as glucocorticoids are often used in the management of SLE. The research was carried out in accordance with the Declaration of Helsinki. The study protocol was approved by the Institutional Review Committee at Hospital Universitario de Canarias and at Hospital Universitario Doctor Negrín (both in Spain), and all subjects provided informed written consent (Approval Number 2015_84).

### 2.2. Data Collection and Laboratory Assessments

The individuals included in the study completed a cardiovascular risk factor and medication use questionnaire and underwent a physical examination. The weight, height, body-mass index, abdominal circumference, and systolic and diastolic blood pressure (measured with the participant in a supine position) were assessed under standardized conditions. Information regarding the smoking status (current smokers) and hypertension treatment was obtained from the questionnaire. The medical records were reviewed to ascertain specific diagnoses and medications. The SLE disease activity and damage were assessed using the systemic lupus erythematosus disease activity index−2000 (SLEDAI-2K) [13] and systemic lupus international collaborating clinics/the American college of rheumatology (SLICC/ACR damage index, SDI) [14], respectively. For the purpose of the present study, the SLEDAI-2k index was divided into none (0 points), mild (1–5 points), moderate (6–10 points), high (11–19), and very high activity (>20) categories as previously described [15]. The disease severity was also measured using the Katz index [16]. The Katz index ranges from 0 to a maximum score possible of 13, and high severity is defined as equal to or higher than 3 points. An ELISA kit was used for the detection of TGF-β1 (Elabscience, Houston, TX, USA). Both the intra and inter-coefficients of variability were <10% for this assay.

### 2.3. Statistical Analysis

The demographics and clinical characteristics of patients with SLE and the controls were described as the mean ± standard deviation (SD) or percentages for the categorical variables. For the non-normally distributed continuous variables, the data were expressed as the median and interquartile range (IQR). The relationship between the features of the disease with circulating TGF-β1 was assessed through a multivariable linear regression analysis showing non-standardized beta coefficients. The confounders were selected from the demographic-related data that had a univariable relationship with TGF-β1 with a *p*-value less than 0.20. All the analyses used a 5% two-sided significance level and were performed using Stata software, version 17/SE (StataCorp, College Station, TX, USA) and *p*-values of <0.05 were considered statistically significant.

## 3. Results

### 3.1. Demographics and Disease-Related Data of Systemic Lupus Erythematosus Patients

The serum levels of TGF-β1 in our series of SLE patients were 3165 ± 2652 pg/mL. These were found to be in the expected range, as previously described in other studies [17,18,19]. A histogram of the frequency distribution of TGF-β1 is shown in Supplementary Figure S1. The demographics and disease-related characteristics of the 284 patients with SLE included in this study are shown in Table 1. Most of them were women (92%), and the mean age ± SD was 50 ± 12 years. The body mass index of the participants was 28 ± 6 kg/m2, and the average abdominal circumference was 92 ± 13 cm. Classic cardiovascular risk factors were not uncommon. For example, 24% of the patients were current smokers, 39% had hypertension, and 30% were obese. Also, 25% of the patients were taking statins, and 29% were taking aspirin (Table 1).

The disease duration was 16 (IQR 7-24) years. Most of the patients with SLE were in the categories of no activity (40%) or mild-moderate activity (39%), as shown by the SLEDAI score. The SLICC and Katz indexes were 1 (IQR 0-2) and 2 (IQR 1-4), respectively. Seventy-eight percent of the patients had a SLICC score equal to or higher than 1. Half of the patients (50%) were taking prednisone, and the median daily dose of prednisone was 5 mg/day (IQR 5-7.5 mg). At the time of recruitment, 67% of patients were found to be positive for anti-DNA, and 69% were positive for ENA, with anti-SSA being the antibody most frequently found (35%). Sixty-nine percent of the patients were taking hydroxychloroquine when the study was performed. Other medications less commonly used were methotrexate (11%) and azathioprine (15%). Additional information on the SLE-related data is shown in Table 1.

### 3.2. Multivariable Analysis of the Relationship of the Demographic and Disease-Related Data with the Serum Levels of TGF-β1

Age, abdominal circumference, and hypertension were significantly related to higher serum levels of TGF-β1 (Table 2). With respect to the SLE-related data, the duration of disease, presence of anti-SSB, and use of mycophenolate mofetil were associated with significantly higher circulating TGF-β1. In the univariable analysis, the Katz severity index, both binary (greater than or equal to 3 points) and continuous, was associated with significantly higher serum levels of TGF-β1. However, after adjusting for confounders, only thisindex as a continuous variable was associated with TGF-β1 (beta coefficient-179 [95% confidence interval -CI- 7–350] pg/mL, *p* = 0.041). Similarly, the SLEDAI index was associated with greater circulating TGF-β1 after the multivariable analysis (beta coefficient 96 [95% CI 20–171] pg/mL, *p* = 0.014). However, when this score was divided into its different categories (no activity, mild, and moderate to very high), no differences were found between the highest compared to the no activity reference category (Table 2). Furthermore, the SLICC score was not related to the serum TGF-β1 levels.

### 3.3. Relationship of Activity Scores, Damage, and Disease Severity with TGF-β1

Since disease scores represent the sum or combination of various aspects of the disease, we broke down these scores and analyzed the relationship of their elements, one by one, with TGF-β1 (Table 3). Regarding the Katz index, a history of proteinuria, a recorded hematocrit of less than 30%, or a serum creatinine level greater than 1.3 mg/dL were significantly and positively associated with higher serum TGF-β1 levels. Surprisingly, although the total SLEDAI score was significantly associated with the highest circulating TGF-β1, when this score was split into different items, none of these items were significantly associated with TGF-β1. Furthermore, while the SLICC domains related to ocular and cardiovascular involvements showed a positive and significant association with TGF-β1, the presence of gastrointestinal and musculoskeletal manifestations was related to significantly lower serum levels of TGF-β1 (Table 3 and Supplementary Figure S2). The full relationship between the SLICC score items and circulating TGF-β1 is additionally shown in Supplementary Table S1.

## 4. Discussion

To our knowledge, our study is the largest in the literature in which TGF-β1 is studied in a series of well-characterized SLE patients with a wide range of disease manifestations. According to our results, TGF-β1 is highly related to various manifestations of the disease. These findings support the role of TGF-β1 in the pathophysiology of SLE.

In previous work with 70 Egyptian patients with SLE [20], the TGF-β1 levels were significantly correlated with hemoglobin, platelet count, and inversely with erythrocyte sedimentation rate, serum creatinine, and urea. Furthermore, significantly lower levels of TGF-β1 were found in patients with high disease activity (SLEDAI > 10), while the level tended to be lower in those with organ damage. In that study, TGF-β1 was significantly lower in patients with serum creatinine >1.2 mg/dL than in those with <1.2 mg/dL, and also in patients with a glomerular filtration rate of <50 mL/min than in those with a glomerular filtration rate of >50 mL/min. However, all the analyses and associations were shown on a univariable basis, and no adjustments for confounders were performed. In another study on 188 patients with SLE stratified according to the presence or absence of lupus nephritis [21], the patients with nephritis showed significantly lower serum TGF-β1 values compared to the patients without lupus nephritis. Furthermore, the lowest serum TGFβ1 levels were found in patients with class V-type nephritis. There were significant negative correlations between TGF-β1 and SLEDAI levels, fever, arthritis, proteinuria, hematuria, serum creatinine, thrombocytopenia, lymphopenia, erythrocyte sedimentation rate, ANA, urinary leukocytes, and cell casts. Lupus nephritis patients showed a positive correlation of TGF-β1 levels with the estimated glomerular filtration rate and C3 and C4 serum levels [21]. However, in this study, the patients were only categorized according to the presence or absence of lupus nephritis, and, as occurred with the series of Egyptian patients with SLE, no statistical adjustments were performed.

The role of TGF-β1 gene variants in SLE susceptibility was described before [9]. In this work, the association of the +869 T > C (rs1800470) and -509 C > T (rs1800469) TGF-β1 variants with susceptibility, autoantibodies, disease activity, and TGF-β1 plasma levels were studied. The TGF-β1+ 869 CC genotype was associated with SLE susceptibility and with a reduction in C4 and TGF-β1 levels. The authors concluded that the TGF-β1+ 869 T > C variant could be used as a genetic marker for SLE susceptibility and some variants as predictors of laboratory activity. In another study, the genetic variants of *FOXP3* (factor forkhead box protein 3) were associated with SLE susceptibility. The G/C haplotype of this gene provided protection for SLE, possibly by increasing the TGF-β1 levels [8]. These two works emphasize the relevance of TGF-β1 genetics and probably its circulating levels in the autoimmunity of SLE.

The multivariate analysis of the data from our series supports a positive relationship between the activity and severity of the SLE with the serum levels of TGF-β1. This positive relationship was somewhat unexpected since TGF-β1 has, in general, an immunosuppressive and beneficial effect in terms of autoimmunity. However, a possible explanation for our findings may be that this molecule may be upregulated as a compensatory mechanism in SLE patients.

Both the positive and negative relationship of TGF-β with some characteristics of SLE disease were previously described [20,21]. Accordingly, when we approached the relationship between the damage index and TGF-β1, we observed a positive relationship between ocular and cardiovascular damage and TGF-β1. In contrast, both gastrointestinal and musculoskeletal manifestations were negatively associated with TGF-β1 levels. In this regard, it is known that the expression of TGF-β1 is context-dependent because TGF-β could contribute to the differentiation of both regulatory (suppressor) and inflammatory T cells. For example, while TGF-β1 production is reduced in some autoimmune diseases, it is increased in many pathological conditions, including pulmonary fibrosis, glomerulosclerosis, renal interstitial fibrosis, cirrhosis, Crohn’s disease, cardiomyopathy, scleroderma, and chronic graft-versus-host disease [1]. Because of this, we think that TGF serum levels may change depending on the predominantly affected organ.

We acknowledge the limitation that we did not include a healthy control group. However, our purpose was to study the serum levels of this molecule in patients with SLE and how it relates to disease characteristics. We also recognize that the influence of the treatments on TGF-β1 cannot be exactly known since our study had a cross-sectional design. Moreover, cumulative exposition to prednisone, hydroxychloroquine, or other treatments was not assessed in our work. Third, in our study, we used a commercially validated ELISA kit for the assessment of TGF-β1 in sera. However, it is known that platelet degranulation could artefact TGF-β1 serum levels. For this reason, future studies in plasma and not sera that take into account the platelet-released pool of TGF- β1 are needed to confirm our results.

## 5. Conclusions

In conclusion, TGF-β1 is highly related to various clinical and laboratory manifestations of SLE. This fact supports the potential role of TGF-β1 in the pathogenesis of SLE. The evaluation of circulating TGF-β1 in SLE may be of interest to identify patients with specific phenotypes of the disease. It may open the possibility of using specific therapies that target this molecule in patients with SLE.

## Figures and Tables

**Table 1:** Characteristics of the SLE patients.

	SLE Patients (*n* = 284)
Age, years	50 ± 12
Female, *n* (%)	261 (92)
Body mass index, kg/m^2^	28 ± 6
Abdominal circumference, cm	93 ± 14
Hip circumference, cm	103 ± 12
Waist-to-hip ratio	0.90 ± 0.07
Systolic pressure, mmHg	127 ± 20
Diastolic pressure, mmHg	79 ± 11
Cardiovascular co-morbidity	
Smoking, *n* (%)	69 (24)
Diabetes, *n* (%)	16 (6)
Hypertension, *n* (%)	111 (39)
Obesity, *n* (%)	85 (30)
Statins, *n* (%)	72 (25)
Aspirin, *n* (%)	80 (29)
SLE-related data	
Disease duration, years	16 (7–24)
CRP, mg/dL	2.0 (0.8–4.4)
SLICC	1 (0–2)
SLICC ≥1, *n* (%)	191 (68)
Katz index	2 (1–4)
Katz ≥3, *n* (%)	126 (44)
SLEDAI	2 (0–4)
SLEDAI categories, *n* (%)	
No activity, *n* (%)	109 (40)
Mild, *n* (%)	107 (39)
Moderate, *n* (%)	41 (15)
High, *n* (%)	10 (4)
Very high, *n* (%)	4 (1)
Auto-antibody profile	
Anti-DNA positive, *n* (%)	151 (67)
ENA positive, *n* (%)	164 (69)
Anti-SSA, *n* (%)	55 (35)
Anti-SSB, *n* (%)	36 (21)
Anti-RNP, *n* (%)	64 (28)
Anti-Sm, *n* (%)	24 (10)
Anti-ribosome	13 (9)
Anti-nucleosome	32 (22)
Anti-histone	22 (15)
Anti-phospholipid syndrome, *n* (%)	43 (16)
Anti-phospholipid autoantibodies, *n* (%)	61 (32)
Lupus anti-coagulant, *n* (%)	51 (28)
ACA IgM, *n* (%)	22 (11)
ACA IgG, *n* (%)	39 (20)
Anti-beta2 glycoprotein IgM, *n* (%)	19 (10)
Anti-beta2 glycoprotein IgG, *n* (%)	28 (15)
C3, mg/dL	130 ± 40
C4, mg/dL	21 ± 12
Current prednisone, *n* (%)	140 (50)
Prednisone, mg/day	5 (5–7.5)
Hydroxychloroquine, *n* (%)	194 (69)
Methotrexate, *n* (%)	31 (11)
Mycophenolate mofetil, *n* (%)	31 (11)
Azathioprine, *n* (%)	43 (15)
Rituximab, *n* (%)	8 (3)
Belimumab, *n* (%)	8 (3)

The data represent the mean ± SD or median (interquartile range) when the data were not normally distributed. C3 C4: complement; CRP: C reactive protein. ACA: anti-cardiolipin. ENA: extractible nuclear antibodies. SLEDAI: Systemic Lupus Erythematosus Disease Activity Index. SLEDAI categories were defined as: 0, no activity; 1–5, mild; 6–10, moderate; >10, high activity; >20, very high activity. SLICC: systemic lupus international collaborating clinics/American colleague of rheumatology damage index.

**Table 2:** Demographics and disease characteristics in relation to the TGF-β1 serum levels.

	TGF-β1, pg/mL			
	Beta Coefficient 95%CI, *p*			
	Univariable		Adjusted *	
Age, years	28 (0.6–56)	0.045		
Female	−1032 (−2165–101)	0.074		
Body mass index, kg/m^2^	46 (−9–102)	0.10		
Abdominal circumference, cm	25 (2–48)	0.034		
Hip circumference, cm	7 (−21–34)	0.64		
Waist-to-hip ratio	7726 (3479–11972)	<0.001		
Systolic pressure, mmHg	−4 (−20–13)	0.66		
Diastolic pressure, mmHg	−7 (−36–22)	0.64		
Cardiovascular co-morbidity				
Smoking	43 (−688–775)	0.91		
Diabetes	1300 (−38–2639)	0.057		
Hypertension	1237 (605–1868)	<0.001		
Obesity	544 (−145–1234)	0.12		
Statins	409 (−317–1135)	0.27		
Aspirin	534 (−174–1242)	0.14		
SLE-related data				
Disease duration, years	72 (41–102)	<0.001	70 (38–102)	<0.001
CRP, mg/dL	14 (−14–41)	0.34		
SLICC	34 (−146–213)	0.71		
SLICC ≥1	−184 (−862–495)	0.59		
Katz index	315 (157–473)	<0.001	179 (7–350)	0.041
Katz ≥3	1069 (445–1693)	<0.001	608 (−41–1256)	0.066
SLEDAI	84 (8–160)	0.031	96 (20–171)	0.014
SLEDAI categories				
No activity	−	−		
Mild	−46 (−776–684)	0.90		
Moderate to very high	228 (−664–1120)	0.62		
Auto-antibody profile				
Anti-DNA positive	−416 (−1193–361)	0.29		
ENA positive	503 (−201–1207)	0.16	465 (−235–1164)	0.19
Anti-SSA	428 (−535–1390)	0.38		
Anti-SSB	2745 (584–4906)	0.013	2478 (400–4557)	0.020
Anti-RNP	−250 (−1008–509)	0.52		
Anti-Sm	−465 (−1552–622)	0.40		
Anti-ribosome	−240 (−1649–1169)	0.74		
Anti-nucleosome	828 (−158–1814)	0.099	936 (−25–1897)	0.056
Anti-histone	828 (−309–1965)	0.15	694 (−422–1810)	0.22
Anti-phospholipid syndrome	336 (−410–1082)	0.38		
Anti-phospholipid autoantibodies				
Lupus anti-coagulant	648 (−159–1456)	0.12	348 (−507–1203)	0.42
ACA IgM	−181 (−1272–910)	0.74		
ACA IgG	245 (−618–1108)	0.56		
Anti-beta2 glycoprotein IgM	822 (−347–1992)	0.17	900 (−259–2059)	0.13
Anti-beta2 glycoprotein IgG	−28 (−1002–946)	0.96		
C3, mg/dL	−3 (−12–5)	0.47		
C4, mg/dL	3 (−25–31)	0.82		
Current prednisone	251 (−384–886)	0.44		
Prednisone, mg/day	−123 (−271–25)	0.10	−34 (−670–602)	0.92
Hydroxychloroquine	3598 (−1625–8821)	0.18	−396 (−1099–307)	0.27
Methotrexate	−651 (−1660–358)	0.21		
Mycophenolate mofetil	1473 (445–2502)	0.005	962 (−82–2066)	0.071
Azathioprine	50 (−827–927)	0.91		
Rituximab	−146 (−2022–1731)	0.88		
Belimumab	816 (−1059–2690)	0.39		

In this analysis, the TGF−β1 serum levels were considered the dependent variable. The beta coefficients are shown as non-standardized data. C3 C4: complement; CRP: C reactive protein. ACA: anti-cardiolipin. ENA: extractible nuclear antibodies. SLEDAI: systemic lupus erythematosus disease activity index. SLEDAI categories were defined as: 0, no activity; 1–5, mild; 6–10, moderate; >10, high activity; >20, very high activity. SLICC: systemic lupus international collaborating clinics/American colleague of rheumatology damage index. * Adjusted for disease duration, age, gender, body mass index, aspirin, and hypertension.

**Table 3:** Disease score items in relation to the TGF-β1 serum levels.

			TGF-β1, pg/mL	
	*n*	%	Beta Coefficient (95%)	*p*
Katz index				
History of cerebritis (seizure or organic brain syndrome)	12	6	406 (−544–1355)	0.40
History of pulmonary disease	10	5	121 (−879–1121)	0.81
Biopsy proven diffuse proliferative glomerulonephritis	23	12	640 (−19–1299)	0.057
4−6 ARA criteria for SLE satisfied to date	139	73	−472 (−1442–498)	0.34
7 or more ARA criteria for SLE satisfied to date	23	12	66 (−586–719)	0.84
History of proteinuria (2+ or more)	62	32	1775 (876–2674)	<0.001
Lowest recorded hematocrit to date = 30−37%	88	46	−593 (−1463–278)	0.18
Lowest recorded hematocrit to date < 30%	47	25	772 (282–1262)	0.002
Highest recorded creatinine to date = 1.3−3	28	15	2286 (1093–3480)	<0.001
Highest recorded creatinine to date > 3	3	2	925 (−772–2623)	0.28
SLEDAI				
Seizures	1	0	−754 (−6058–4549)	0.78
Psychosis	1	0	2382 (−2914–7679)	0.38
Organic brain syndrome	0	0	−	−
Visual disturbance	1	0	−1532 (−6834–3769)	0.57
Cranial nerve disorder	1	0	−1844 (−7143–3456)	0.49
Lupus headache	1	0	−1632 (−6934–3668)	0.55
ACVA	0	0	−	−
Vasculitis	1	0	−1949 (−7248–3350)	0.47
Arthritis	9	3	−984 (−2686–899)	0.33
Myositis	0	0	−	−
Urinary cylinders	7	3	−918 (−2945–1109)	0.37
Hematuria	16	6	−1120 (−2480–240)	0.11
Proteinuria	5	2	1884 (−497–4265)	0.12
Pyuria	11	4	−231 (−1863–1401)	0.78
Rash	21	8	−730 (−1931–471)	0.23
Alopecia	11	4	−954 (−2581–672)	0.25
Mucosal ulcers	14	5	205 (−1249–1659)	0.78
Pleurisy	3	1	−1654 (−4713–1422)	0.29
Pericarditis	1	0	−1467 (−6769–3834)	0.59
Low complement	76	28	159 (−576–894)	0.67
Elevated anti-DNA	85	31	637 (−67–1341)	0.076
Fever	2	1	−591 (−4349–3166)	0.76
Thrombopenia	10	4	899 (−805–2602)	0.30
Leukopenia	19	7	−998 (−2254–257)	0.12
SLICC domains				
Ocular	63	22	875 (128–1623)	0.022
Neuropsychiatric	40	14	768 (−152–1688)	0.10
Renal	28	10	584 (−474–1642)	0.28
Pulmonary *	19	7	−457 (−1700–785)	0.47
Cardiovascular	23	8	1442 (316–2569)	0.012
Peripheral vascular	34	12	−796 (−1750–157)	0.10
Gastrointestinal	28	10	−1119 (−2171–68)	0.037
Musculoskeletal	89	31	−1548 (−2199– -897)	<0.001
Skin	39	14	−635 (−1536–266)	0.17
Premature gonadal failure	19	7	1553 (287–2818)	0.016
Diabetes (regardless of treatment)	16	6	990 (−284–2264)	0.13
Malignancy (excluded dysplasia)	11	4	−81 (−1696–1533)	0.92

* History of pulmonary disease refers to the presence of lupus pneumonitis, pulmonary hemorrhage, or pulmonary hypertension. SLEDAI: systemic lupus erythematosus disease activity index; SLE: systemic lupus erythematosus. SLICC: systemic lupus international collaborating clinics/American colleague of rheumatology damage index. The presence of a SLICC domain is shown if the items point in the domain are ≥1 (see Supplementary Table S1). The beta coefficients are shown as non-standardized data. ARA: American rheumatism association; ACVA: acute cerebrovascular accident.

## Data Availability

The data sets used and analyzed in the present study are available from the corresponding author upon request.

## Supplementary Materials

The following supporting information can be downloaded at: https://www.mdpi.com/article/10.3390/biom13010073/s1, Figure S1: Histogram of frequency distribution of TGF-β1 serum values in SLE patients; Figure S2: Histogram of frequency distribution of SLE patients without (left) and with (right) musculoskeletal manifestations (beta coefficient-1548 (-2199-897) pg/mL, *p* < 0.001). Table S1: Relation of SLICC score items to TGF-β1.
