# Supplementary material for: Serum Levels of Transforming Growth Factor Beta 1 in Systemic Lupus Erythematosus Patients
Source: Biomolecules. 2022 Dec 29;13(1):73. doi: 10.3390/biom13010073 (PMC9855909; doi:10.3390/biom13010073)
Supplement: Supplementary file 1 [file biomolecules-13-00073-s001.zip › biomolecules-2006031-supplementary.pdf]

Supplementary Table S1. Relation of SLICC score items to TGF-β1.

	n	%	TGF-β1, pg/ml<br>beta coef. (95%)	p
Ocular				
Any cataract ever	29	11	1122 (74-2171)	0.036
Retinal change or optic atrophy	33	12	1713 (755-2672)	0.001
Points =>1 in the domain	63	22	875 (128-1623)	0.022
Neuropsychiatric				
Cognitive impairment	7	3	-104 (-2116-1908)	0.92
Seizures requiring therapy for 6 months	15	5	993.2 (-444-2430)	0.18
Cerebrovascular accident ever	13	5	476 (-1074-2027)	0.55
Cranial or peripheral neuropathy	5	2	4321 (1726-6915)	0.001
Transverse myelitis	1	0	3167 (-2082-8416)	0.24
Points =>1 in the domain	40	14	768 (-152-1688)	0.10
Renal				
Estimated or measured glomerular filtration rate <50%	13	5	2538 (1016-4060)	0.001
Proteinuria 3.5 gm/24 hours	7	3	-1204 (-3212-804)	0.24
End-stage renal disease	4	1	-126 (-1007-756)	0.78
Points =>1 in the domain	28	10	584 (-474-1642)	0.28
Pulmonary				
Pulmonary hypertension	4	1	2257 (-373-4887)	0.092
Pulmonary fibrosis	4	1	-1312 (-3952-1327)	0.33
Shrinking lung	2	1	-1769 (-5488-1951)	0.35
Pleural fibrosis	1	0	-633 (-5890-4625)	0.81
Pulmonary infarction	1	0	-1220 (-6484-4043)	0.65
Points =>1 in the domain	19	7	-457 (-1700-785)	0.47
Cardiovascular				
Angina or coronary artery bypass	4	1	-685 (-3335-1965)	0.61
Myocardial infarction ever	2	1	-1200 (4932-2532)	0.53
Cardiomyopathy	2	1	4543 (849-8238)	0.02
Valvular disease	9	3	4216 (2505-5926)	<0.001
Pericarditis for 6 months, or pericardiectomy	2	1	-699 (-4433-3035)	0.71
Points =>1 in the domain	23	8	1442 (316-2569)	0.012
Peripheral vascular				
Claudication for 6 months	3	1	-550 (-3633-2532)	0.73
Minor tissue loss (pulp space)	5	2	-1085 (-3478-1309)	0.37
Significant tissue loss ever	0	0	-	-
Venous thrombosis	14	5	-369 (-1827-1089)	0.62
Points =>1 in the domain	34	12	-796 (-1750-157)	0.10
Gastrointestinal				

Infarction or resection of the "terminal" duodenum, spleen, liver or gallbladder for any reason	22	8	-1078 (-2266-110)	0.075
Mesenteric insufficiency	1	0	-1913 (-7175-3349)	0.48
Chronic peritonitis	1	0	978 (-4288-6244)	0.72
Stricture or upper gastrointestinal tract surgery ever	0	0	-	-
Pancreatic insufficiency	0	0	-	-
Points =>1 in the domain	28	10	-1119 (-2171-68)	0.037
Musculoskeletal				
Muscle atrophy or weakness	3	1	-1247 (-4337-1842)	0.43
Deforming or erosive arthritis	40	15	-1446 (-2354-(-539))	0.002
Osteoporosis with fracture or vertebral collapse	23	9	-671 (-1832-490)	0.26
Avascular necrosis	7	3	-400 (-2440-1641)	0.70
Osteomyelitis	1	0	-1597 (-6930-3736)	0.56
Tendon rupture	4	2	-1944 (-4617-729)	0.15
Points =>1 in the domain	89	31	-1548 (-2199-897)	<0.001
Skin				
Scarring chronic alopecia	16	6	-191 (-1543-1162)	0.78
Extensive scarring or panniculitis	10	4	-1931 (-3605-(-256))	0.024
Skin ulceration	4	1	-370 (-3016-2277)	0.78
Points =>1 in the domain	39	14	-635 (-1536-266)	0.17
Premature gonadal failure	19	7	1553 (287-2818)	0.016
Diabetes (regardless of treatment)	18	6	990 (-284-2264)	0.13
Malignancy (exclude dysplasia)	11	4	-81 (-1696-1533)	0.92

SLICC items and domains represent the independent variable.
SLICC: Systemic Lupus International Collaborating Clinics/American Colleague of Rheumatology Damage Index.
Beta coefficients are shown non-standardized

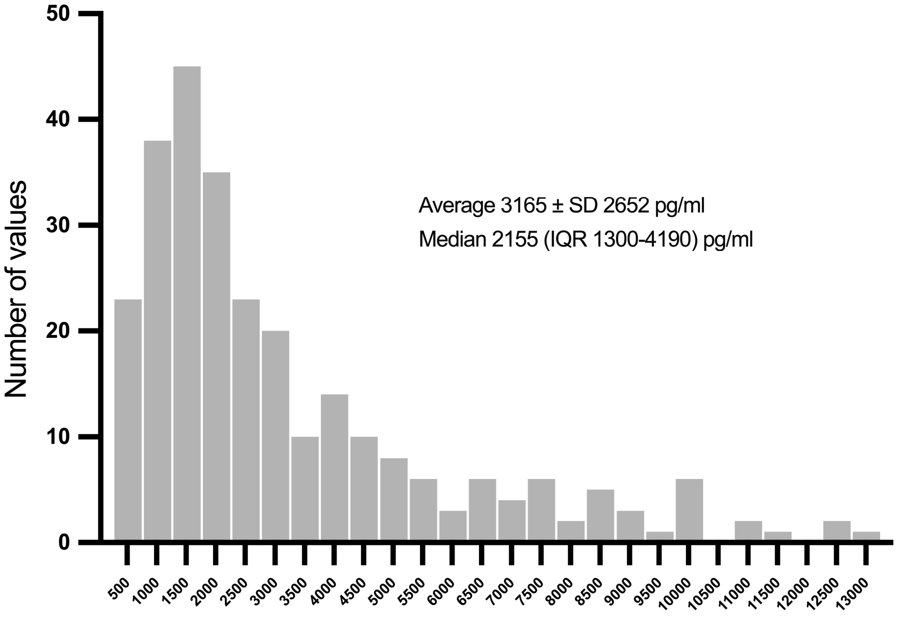

Supplementary Figure S1. Histogram of frequency distribution of TGF-β1 serum values in SLE patients.

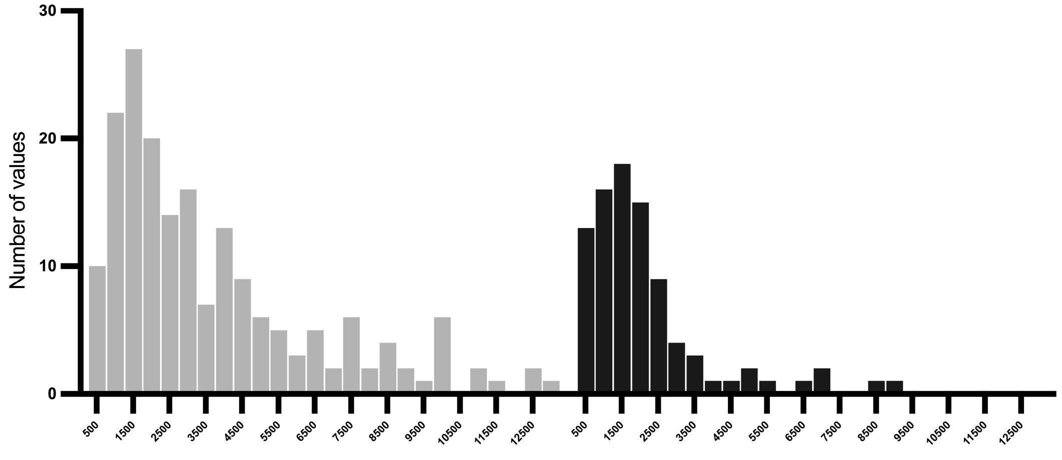

Supplementary Figure S2. Histogram of frequency distribution of SLE patients without (left) and with (right) musculoskeletal manifestations (beta coefficient -1548 (-2199-897) pg/ml, $p<0.001$).
